# Adolescents encouraging healthy lifestyles through a peer‐led social marketing intervention: Training and key competencies learned by peer leaders

**DOI:** 10.1111/hex.13406

**Published:** 2021-12-22

**Authors:** Elisabet Llauradó, Magaly Aceves‐Martins, Jordi Prades‐Tena, Maria Besora‐Moreno, Ignasi Papell‐Garcia, Montse Giralt, Amy Davies, Lucia Tarro, Rosa Solà

**Affiliations:** ^1^ Facultat de Medicina i Ciències de la Salut, Functional Nutrition, Oxidation, and Cardiovascular Diseases Group (NFOC‐Salut) Universitat Rovira i Virgili Reus Spain; ^2^ Institut d'Investigació Sanitària Pere Virgili Reus Spain; ^3^ Departament d'Estudis de Comunicació Universitat Rovira i Virgili Tarragona Spain; ^4^ Unitat de Nutrició i Salut, Eurecat Centre Tecnològic de Catalunya Reus Spain; ^5^ Health & Social Care National Children's Bureau London UK; ^6^ Hospital Universitari Sant Joan de Reus Reus Spain

**Keywords:** adolescent, education, health education, health promotion, lifestyle

## Abstract

**Background:**

Adolescents who participate as peer leaders can benefit and acquire competencies from their peer leadership experience.

**Objectives:**

To identify the competencies gained by adolescents who participated as peer leaders in a healthy lifestyle study and to determine whether the training characteristics were related to improvement in competencies.

**Design:**

The present study was part of the European Youth Tackling Obesity (EYTO) project, a multicentre social marketing intervention involving four European countries.

**Setting and Participants:**

Eighteen peer leaders (aged 13–15 years, three or five leaders per country) from disadvantaged neighbourhoods received training in designing and implementing activities for their peers.

**Measures:**

The peer leaders' confidence, experience and interest in 11 tasks related to lifelong learning competencies were assessed with questions rated on a colour scale at baseline and at the end of the study.

**Results:**

The peer leaders demonstrated improvements in experience, confidence and interest in different tasks, such as research, website or logo design, oral presentations, social media use and collaboration with people from other countries. They increased their confidence in management tasks (*p* = 0.03) and their confidence and experience in communication tasks (*p* = 0.01). The peer leaders from Spain and Portugal had greater improvements than those from the other countries.

**Conclusion:**

The peer leaders improved their confidence in management tasks and their confidence and experience in communication tasks. Slight differences were detected in improvement in competencies by country, likely due to the differences in the peer training applied. Recommendations for peer leader training are proposed, although these results should be verified with larger sample size.

**Patient or Public Contribution:**

The peer leaders contributed to the design and implementation of the training and intervention.

## 1 INTRODUCTION

Risk behaviours adopted during adolescence influence noncommunicable diseases in adulthood.^1^ For this reason, investment in adolescents' capabilities is vital for the UN's Sustainable Development Agenda, considering that investment in health and education generates a large economic and social return and transforms adolescents' lives.^2^

Encouraging healthy lifestyles among adolescents is a challenge that requires the successful implementation of educational methodological strategies^3^ because, at this stage, healthy lifestyles are affected not only by family influences but also as they are in preschoolers; for example, school and peer influences on lifestyles become more important during the childhood and adolescence.^4^ In this context, peer‐led programmes have emerged as an educational strategy that involves the effective transmission of information and behaviours between peer leaders as educators and their peers.^5^ As a result, adolescent peer leaders solve their own issues, support their peers^6^ and act as lifestyle mentors through actions performed in their daily lives.^7^ In particular, youth peer‐led interventions have shown greater positive results than adult‐led interventions with respect to improving the diets of and/or the amount of physical activity^8^, ^9^, ^10^ of youths in the promotion of healthy lifestyles and the prevention of risky behaviours.^11^, ^12^, ^13^ To demonstrate the effectiveness of peer‐led programmes designed to improve the lifestyles of adolescents, it is necessary to elucidate the skills and competencies achieved during training by peer educators.^14^ Moreover, this type of programme goes beyond changing only lifestyles, having been demonstrated to increase student connectedness to the self, others and school for both peer educators and peer education receivers.^14^ In addition, this increase in connectedness has been associated with academic performance improvement.^15^

A peer leader is a person who shares similar status and experiences to those whom they are leading,^16^ and can be considered a health educator after training. According to the World Health Organization (WHO), a health educator assesses community needs; plans, implements and assesses the effectiveness of health education programmes; coordinates the provision of health education services; acts as a resource person in health education; and reinforces health communication.^17^ Peer leaders create a favourable environment and implement different tactics or strategies to promote student participation and facilitate sessions.^18^ Peer leaders can carry out interventions with similar fidelity to adult educators, as demonstrated in the Peer‐Led Asthma Self‐Management Program for Adolescents (PLASMA),^19^ and the students who attended peer‐led training demonstrated significant improvements in basic first aid skills, such as cardiopulmonary resuscitation and defibrillation.^20^ The results obtained in the PLASMA study, are not only for the recipients of the training; for example, peer leaders who participated in this study improved their knowledge about asthma, their quality of life and their control about asthma.^21^ Although there have been published studies and systematic reviews showing improvements in communication skills, confidence and knowledge,^20^, ^21^, ^22^, ^23^ only some of them presented results about the benefits of being a peer leader.^24^, ^25^ For this reason, more studies are needed to delve into the benefits for leaders.

A peer‐led strategy among adolescents was the methodological keystone of the European Youth Tackling Obesity (EYTO) project, a multicentre social marketing intervention that involved the United Kingdom (UK), Spain, Portugal and the Czech Republic (CZ). Each of the four participating countries acted autonomously and recruited five peer leaders from low‐income neighbourhoods. These leaders designed and implemented activities for interventions focusing on encouraging healthy lifestyles among their high school adolescent peers over a period of 2 academic years, spanning 12 months. All four interventions had five requirements^10^: (1) encourage healthy lifestyles; (2) implement social marketing as a methodological base; (3) implement a peer‐led educational strategy; (4) involve young people in the decision‐making processes; and (5) use social media as the main communication channel among adolescents. Since each country acted autonomously despite having the same five requirements, four separate interventions were developed, with differences in type, place and/or social media used.

In this context, the hypothesis was that the training of peer leaders would influence their competencies acquired during the training process and the implementation of the intervention. Thus, the purpose of the present study was to identify the competencies achieved by adolescents who participated as peer leaders by designing and implementing activities that encouraged healthy lifestyles among their peers over a period of 12 months in the four EYTO participant countries. Additionally, the study aimed to determine whether the training characteristics were related to the improvement of the competencies of peer leaders, to make recommendations for effective adolescent peer leader training and to propose interventions that encourage healthy lifestyles.

## 2 MATERIALS AND METHODS

The present study was part of the EYTO project, a multicentre social marketing intervention involving four European countries: the UK, Spain, Portugal and CZ. The project followed the principles of the Declaration of Helsinki and the Good Clinical Practice Guidelines of the International Conference on Harmonization (ICH GCP). Portugal, CZ and the UK did not conduct any scientific assessment. However, Spain completed a study based on a parallel‐cluster, randomized, controlled, school‐based, peer‐led social marketing intervention that was approved by the Ethics Committee (ref: 14–04–24/4proj2) and was registered at clinicaltrials.gov (NCT02157402).^10^

### 2.1 Subjects

The initial study sample of peer leaders consisted of 20 adolescents aged 13–15 years old who lived in disadvantaged neighbourhoods. The sample included five adolescents from each of the four countries who had been trained as peer leaders to implement the EYTO project and had been given the title of an adolescent challenge creator.^10^

The peer leaders were selected from a youth centre, a scout meeting, a community event and high schools depending on the country from which they were recruited (considering the availability and expertise of each country participant), that is, the UK, Portugal, CZ or Spain, respectively. The inclusion criteria were based on the knowledge of the head of the scout group, communication experts or high school teachers regarding the leadership characteristics and enthusiasm of the students that would support their involvement in a project of this nature. Additionally, English language skills were important since peer leaders needed to be able to communicate with peer leaders from other countries.^10^

### 2.2 Peer leader training

**Figure 1:**
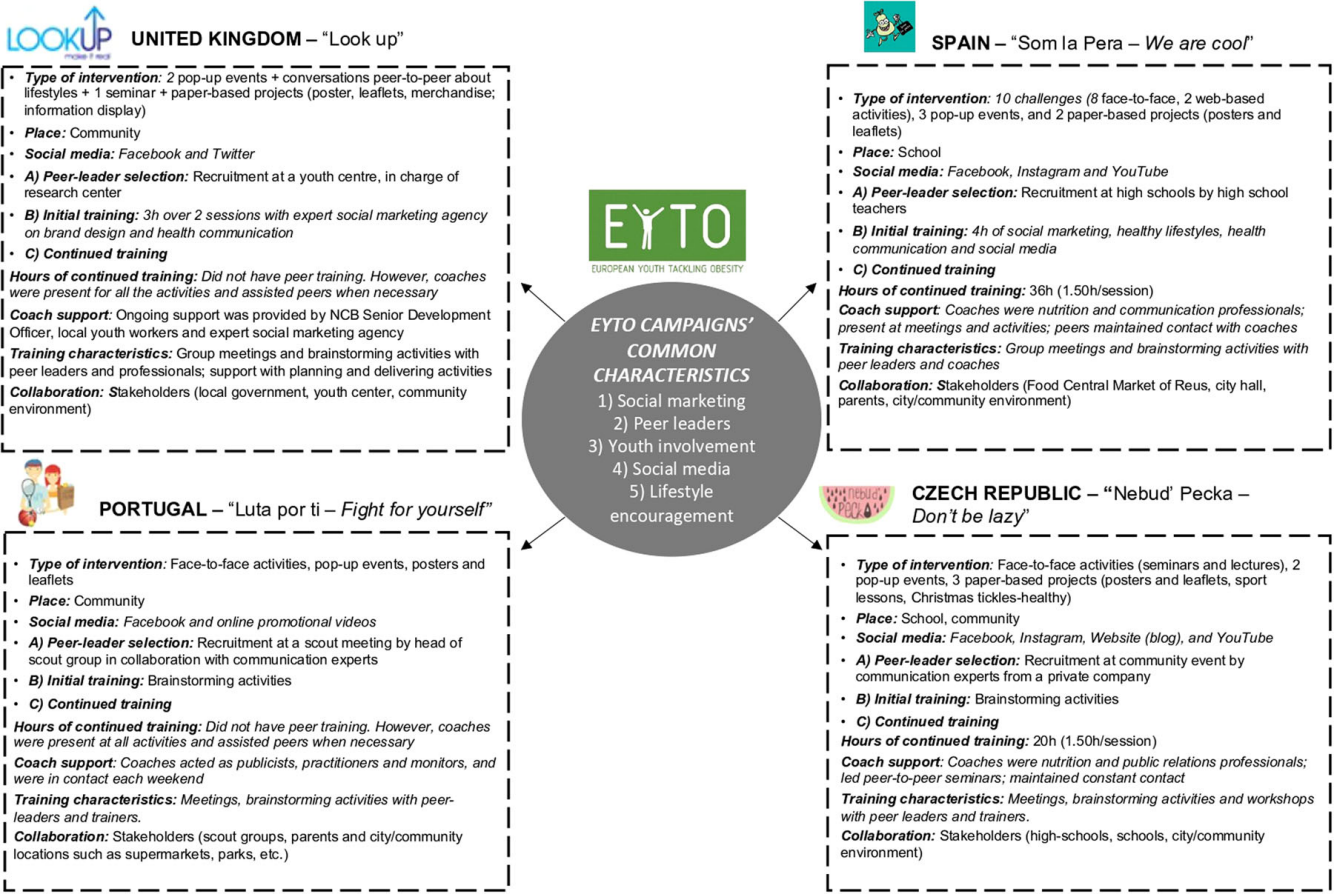
Peer leader training characteristics of adolescents in each country

The training process of the peer leaders was designed according to the Association of American Colleges and Universities (AAC&U) and included the acquisition of adolescent skills through frequent interactions with peer coaches and other peer leaders.^26^ The EYTO training consisted of two stages over 12 months, as shown in Figure 1.

(1) Peer leaders' initial training: CZ and Portugal developed brainstorming activities to begin the design of activities. In contrast, the training of peer leaders from Spain focused on education about healthy lifestyles, health communication through social media and social marketing for 4 h. For peer leaders from the UK, a social marketing agency developed the initial training based on building adolescent communication skills and self‐confidence. This training was presented in two 3‐h sessions.
(2) Peer leaders' continued training:

a. Hours of continued training: Peer leader training ranged from 0 to 36 h in the participating countries. In the UK and Portugal, peer leader training sessions were performed only as support strategies when the peer leaders requested them. In contrast, in Spain, to support peer leaders during the design and implementation processes, biweekly peer leader sessions, which were 1.5 h in duration and continued over 24 weeks (36 h of training), were mandated. In CZ, the peer leaders met for 20 h, with weekly sessions of 1.5 h over 13 weeks.
b. Coach support: The role of the coaches was to guide the peer leaders to ensure the fulfilment of the five requirements of the intervention while incorporating the peer leaders' preferences for training, without a predetermined training schedule. The coaches were experts and professionals, such as practitioners, monitors, publicists and communication professionals, public relations professionals and nutrition professionals, from each of the participating countries, as shown in Figure 1. In the UK, the coaches supported the peer leaders only when the peer leaders requested it. In Portugal, the peer leaders were in contact with the coaches every weekend, whereas, in Spain and CZ, the peer leaders and coaches were in constant contact.
c. Peer leader training characteristics: Brainstorming techniques and group meetings encouraging creativity among peer leaders were applied during the initial and continued trainings in each of the four countries. In addition, CZ also held workshops.
d. Collaboration: In all of the countries, stakeholder involvement was similar and focused on city/community environments, such as youth centres, local theatres, public libraries, local markets, academics, private sector stakeholders, policymakers, parents of adolescents and teachers. Additionally, the school environment was incorporated in CZ and Spain.

### 2.3 Data collection: Peer leader training and learning competencies

Initially, two focus groups were conducted based on a guided interview using qualitative semistructured questions. This guided interview allowed for the collection of data regarding the perceptions, attitudes, experiences and beliefs of the participants.^27^ Additionally, in the focus groups, the peer leaders were questioned about what they had gained in terms of self‐confidence, communication skills, and marketing and organization skills, as well as what they had learned regarding healthy lifestyles and encouraging others to follow them.^28^ During the focus groups, which were conducted by external project researchers, the 20 peer leaders (three or five leaders from each of the participating countries) were interviewed in their respective countries at baseline (June 2014) and at the end of the project (December 2015). The general results of the focus groups were published in the final evaluation report of the EYTO project.^28^

The data assessed in the present study were based on the skills of the peer leaders identified in the focus group and included their degree of confidence in developing tasks, their experience and knowledge required to complete the tasks, and their degree of interest in developing and implementing the tasks.^29^ The data led to the identification of 11 specific tasks related to the key competencies that all individuals need for personal fulfilment, development, employment, social inclusion and active citizenship.^29^ Accordingly, the 11 specific tasks to assess the peer leaders' skills were created based on the WHO health educators' definitions as follows^17^: (1) organize an event, for example, a cooking contest, exhibition or gymkhana; (2) evaluate the event activities; (3) design a poster; (4) design a website; (5) design a logo; (6) design a graphic; (7) draft written material to be disseminated to many people; (8) give an oral presentation to a large audience; (9) use social media to communicate a message (through texts, pictures, videos, posters and other formats); (10) engage in a project with unfamiliar people and (11) work with people from other countries.

The quantitative data regarding the 11 tasks were obtained by asking peer leader‐specific questions (Table S1). With respect to the evaluation, each of the 11 tasks was presented in a questionnaire administered to the peer leaders, whereby the peer leaders used stickers to indicate their levels of confidence, experience and interest; specifically, green indicated yes or very much, yellow indicated yes or somewhat and red indicated no or not at all. The results only included the adolescents who answered the questions with a green sticker.

Moreover, from the 11 tasks mentioned above, three different subgroups of tasks were assessed and analysed: (1) management, which included task numbers 1, 2, 7 and 10; (2) design, which included task numbers 3, 4, 5 and 6 and (3) communication, which included task numbers 8, 9 and 11.

### 2.4 Statistical analysis

Categorical variables are presented as percentages (%), and continuous variables are presented as the mean ± standard deviation (±SD). Fisher's exact test was used, due to the small sample size, to detect significant changes in outcomes (confidence, experience and interest) from baseline to the end of the study for each of the 11 project tasks.^30^ Additionally, the *t*‐test was used to detect significant changes in the task subgroups (management, design and communication). All of the results were separated according to confidence, experience and interest based on the baseline versus end‐of‐study results and were independently analysed. All of the analyses were performed using IBM SPSS statistics software, version 27.

## 3 RESULTS

Although 20 peer leaders were recruited to participate in the EYTO project, two peer leaders from the UK did not finish the study because they decided not to continue participating in the EYTO study. Hence, the end‐of‐study analyses included 18 peer leaders from the four participating countries, specifically, five each from Spain, CZ and Portugal and three from the UK.

### 3.1 Experience, confidence and interest of peer leaders in the 11 tasks

Table 1 presents comparisons between baseline and end‐of‐study data regarding peer leaders' confidence, experience and interest in participating in the project based on their evaluations of the 11 tasks. Experience, for example, improved for three tasks: (1) logo design (16.7%–66.7%; *p* = 0.006); (2) oral presentations (72.2%–100%; *p* = 0.045) and (3) social media use (16.7%–72.2%; *p* = 0.002). Confidence improved for four tasks: (1) assessment of activities (64.7%–100%; *p* = 0.008); (2) website design (27.8%–66.7%; *p* = 0.04); (3) social media use (33.3%–72.2%; *p* = 0.04) and (4) collaboration with people from other countries (66.7%–100%; *p* = 0.02). Finally, interest in oral presentations improved (50%–94.4%; *p* = 0.007) (Table 1).

**Table 1:** Peer leaders' experience, confidence and interest in realizing specific tasks

Tasks		Experience			Confidence			Interest		
	Year	*n*	%	*p*‐value*	*n*	%	*p*‐value*	*n*	%	*p*‐value*
1. Organize an event	Baseline	10	55.6	0.15	13	76.5	0.18	18	100	—
	End of study	15	83.3		17	94.4		18	100	
2. Research: Evaluate activities	Baseline	6	33.3	0.32	11	64.7	**0.008**	12	66.7	1.00
	End of study	10	55.6		18	100		13	72.2	
3. Design a poster	Baseline	6	33.3	1.00	11	61.1	0.74	11	61.1	0.12
	End of study	6	33.3		9	50		16	88.9	
4. Design a website	Baseline	3	16.7	0.15	5	27.8	**0.04**	9	50	0.08
	End of study	8	44.4		12	66.7		15	83.3	
5. Design a logo	Baseline	3	16.7	**0.006**	6	33.3	0.09	17	94.4	0.60
	End of study	12	66.7		12	66.7		15	83.3	
6. Prepare a graphic	Baseline	5	27.8	0.49	1	5.6	0.18	7	38.9	0.18
	End of study	8	44.4		5	27.8		12	66.7	
7. Draft written material	Baseline	8	44.4	0.09	6	33.3	0.18	14	77.8	0.66
	End of study	14	77.8		11	61.1		16	88.9	
8. Give an oral presentation	Baseline	13	72.2	**0.045**	10	55.6	0.15	9	50	**0.007**
	End of study	18	100		15	83.3		17	94.4	
9. Use social media	Baseline	3	16.7	**0.002**	6	33.3	**0.04**	15	83.3	1.00
	End of study	13	72.2		13	72.2		14	77.8	
10. Carry out a project	Baseline	9	50	0.31	14	77.8	0.10	15	83.3	0.60
	End of study	13	72.2		18	100		17	94.4	
11. Work with people from other countries	Baseline	9	50	0.50	12	66.7	**0.02**	17	94.4	1.00
	End of study	12	66.7		18	100		18	100	

*Note*: The results show only the percentage of adolescents who answered green to the question. The p‐value of the significant results (*p* < 0.05) is highlighted in bold.

* Fisher's exact test—difference between baseline and end of study values.

Regarding experience, peer leaders from two countries demonstrated some growth. For example, peer leaders from Spain presented increased experience in using social media (0%–100%; *p* = 0.008), while those from Portugal demonstrated increased experience in website design (0%–80%; *p* = 0.048) and logo design (20%–100%; *p* = 0.048), as well as oral presentations (0%–100%; *p* = 0.008). Conversely, peer leaders from the UK and CZ did not significantly increase their experience in any specific task. With respect to confidence at the country‐by‐country level, Spain's peer leaders demonstrated growth in website design (20%–100%; *p* = 0.048) and social media use (20%–100%; *p* = 0.048), whereas peer leaders from CZ exhibited improvements in research skills (20%–100%; *p* = 0.048). However, peer leaders from the UK and Portugal did not reveal any significant improvements. Finally, peer leaders from Spain showed increased interest in website design (0%–100%; *p* = 0.008) and graphic design (0%–100%; *p* = 0.008), whereas those from Portugal exhibited increased interest in oral presentation (20%–100%; *p* = 0.048). Conversely, Portugal demonstrated decreased interest in graphic design (100%–20%; *p* = 0.048) (Table S2).

Table 2 shows the peer leaders' experience, confidence and interest in the three task subgroups, such as management, design and communication tasks. The peer leaders' experience improved for the communication tasks (1.25 ± 1.12 to 2.15 ± 1.09; *p* = 0.01). Their confidence improved for management (2.20 ± 1.58 to 3.20 ± 1.24; *p* = 0.03) and communication (1.40 ± 1.10 to 2.30 ± 1.03; *p* = 0.01) tasks. Finally, their interest did not increase significantly for any subgroup of tasks.

**Table 2:** Peer leaders' experience, confidence and interest in management, design and communication

Subgroups		Experience			Confidence			Interest		
	Year	Mean	SD	*p*‐value*	Mean	SD	*p*‐value*	Mean	SD	*p*‐value*
Management	Baseline	1.65	1.60	0.06	2.20	1.58	**0.03**	2.95	1.47	0.57
	End of study	2.60	1.47		3.20	1.24		3.20	1.28	
Design	Baseline	0.85	1.31	0.08	1.15	1.31	0.11	2.20	1.54	0.17
	End of study	1.70	1.63		1.90	1.55		2.90	1.59	
Communication	Baseline	1.25	1.12	**0.01**	1.40	1.10	**0.01**	2.05	1.05	0.23
	End of study	2.15	1.09		2.30	1.03		2.45	1.00	

*Note*: The results show only the percentage of adolescents who answered green to the question. The p‐value of the significant results (*p* < 0.05) is highlighted in bold.

Abbreviation: SD, standard deviation.

* Student Test —difference between baseline and end of study values.

Furthermore, the subgroup results by country demonstrated that peer leaders from Portugal increased their experience in design (0.40 ± 0.89 to 2.00 ± 0.71; *p* = 0.01) and communication (0 ± 0 to 1.20 ± 0.45; *p* = 0.004) tasks, whereas those from Spain showed improvement only in communication tasks (1.60 ± 0.55 to 3.00 ± 0; *p* = 0.005). The peer leaders from the UK and CZ did not show any significant improvements. Additionally, peer leaders from CZ and Spain increased their confidence in management (1.40 ± 1.34 to 3.40 ± 0.89; *p* = 0.02) and communication (2.20 ± 0.45 to 3.00 ± 0; *p* = 0.02) tasks, respectively. Finally, the peer leaders from Spain (1.60 ± 0.55 to 4.00 ± 0; *p* < 0.001) demonstrated increased interest in design tasks, while those from Portugal (4.00 ± 0 to 3.20 ± 0.45; *p* = 0.02) showed decreased interest in the same tasks (Table S3).

### 3.2 Project evaluation

Moreover, based on the results of the peer leaders' evaluations of their acquired skills conducted in the focus groups, all 18 peer leaders had similar positive opinions about the various benefits of the project. For example, the peer leaders reported gaining self‐confidence, enhancing their communication skills, acquiring marketing and organizational skills, and learning about healthy lifestyles. In particular, the peer leaders reported that they learned how to encourage other people to follow them, and in so doing, they had fun, met new individuals and experienced new cultures.

## 4 DISCUSSION

The present study indicates that adolescents who participated as peer leaders in an intervention to encourage healthy lifestyles among their peers acquired confidence in management tasks and confidence and experience in communication tasks.

Thus, an effective process for training adolescent peer leaders can both improve healthy lifestyles, as demonstrated in Spain's school‐based intervention,^31^ and identify key competencies for adolescents to self‐develop, specifically four of the eight key competencies defined for lifelong learning: (1) communication in the mother tongue; (2) communication in foreign languages; (3) digital competencies and (4) initiative and entrepreneurship competencies.^29^ Peer‐led implementation in other populations, such as medical students, showed that explaining concepts to peers improves the communication skills of peer educators,^32^ is an important skill for working in groups and is one of the emphasized skills for jobs in the digital age.^33^ Adolescence is a good time to learn certain skills, for example, communication because it is known that nonverbal and verbal communication techniques will be important for adolescents' futures in job interviews.^34^ Additionally, better communication skills and free time management are also associated with greater motivation in students.^35^

The peer leader key competencies assessed in this study were more accurate than those reported in other health interventions that evaluated only the skills gained by the participants.^27^ For example, the American nonprofit NutriBee, which performed a community‐based high school adolescent peer leader (13–18 years old) online nutrition intervention, encouraged 10 healthy diet behaviours in middle school students (10–12 years old).^27^ NutriBee peer leaders improved their skills, such as helping others while doing what they enjoyed and being a resource and coach; at the same time, they further developed their personal interests and career paths.^36^ Additionally, it was shown that peer leaders who participated in peer‐led team learning in an engineering course improved their self‐confidence and interest in teaching, although at the beginning of the course, they were apprehensive, worried and unsure.^37^ These acquired competencies were similar to those of the peer leaders in the EYTO project; however, the EYTO project achieved broader results. To our knowledge, the literature on skills gained by peer leaders trained as health educators is scarce. In this context, in one study, peer leaders reported achieving communication, organizational, time management and presentation skills due to their peer‐advising experience. Moreover, the authors of this publication commented that the peer leaders improved their ability to make presentations, conduct individual advising sessions, engage in public speaking and work with others.^38^ However, the study did not include the necessary data to confirm these results. Therefore, it is impossible to compare our results with this study. In a recent study, peer education revealed a confidence improvement of ongoing support on peer leader adolescents while they were motivated to make healthy lifestyle choices and to share their knowledge and experience.^39^ The results of this mentioned study showed similar results to the present study. However, both studies have the same limitation: the small sample size of adolescents acting as peer leaders. Most of the literature has shown results on improvements in lifestyle, as Muzafar presented, with peer leader adolescents significantly improving in some nutritional habits.^40^ However, the previous study did not present results on the skills and competencies of peer leaders.^40^ Additionally, the literature has suggested that to be an effective educator, such as a peer leader or health educator, strong communication skills in combination with knowledge of the subject and enthusiasm to teach are necessary.^41^ For this reason, it is important to know which skills are improved by peer‐leaders related to the improvement of their experience, confidence, and interest of the management tasks and to design guidelines to emphasize the improvement of peer leaders' skills in future interventions.

Interventions focusing on improving lifestyles or health led by peer leaders could provide more benefits than other types of interventions led by adults, and peer leaders could improve their health while spreading health messages. Future interventions should include young people in the dissemination and codesign of the intervention.^42^

Focusing on the experience, confidence and interest of peer leaders in completing their tasks related to management, communication and design, the results indicate that peer leaders' improvements in these areas varied among the four countries. When comparing the results by country, the improvements varied slightly; while the results were similar, the improvement in tasks among the peer leaders from Spain and Portugal was greater than that among the peer leaders from the other countries. Thus, confidence, compared to experience and interest, was the aspect most improved by the peer leaders in the overall and country analyses. Moreover, the peer leaders from Portugal and Spain also presented increased experience in design and communication tasks, while interest in design tasks significantly improved among the peer leaders from Spain, and interest in communication tasks, such as oral presentation, significantly improved among the peer leaders from Portugal.

These differences regarding the key competencies learned could be explained by the dissimilarities among the people who selected the participants, type of setting (such as high school/educational or community), type of activities or interventions, number of hours of training, types of coach support received by the peer leaders, participation in the design of the intervention, materials used and initial English level since these factors represented major differences among the four countries with respect to peer leader training. The evidence indicates that peer leaders should be selected based on their ability to teach, extract ideas and identify concerns, find solutions, facilitate open discussions, be a positive and healthy role model, be open‐minded and be able to supervise and help their high school peers.^43^ In addition, the peer leader selection process should be credible and transparent to peers such that those who were selected are class leaders who demonstrate leadership qualities and who work effectively with teachers and other responsible and involved adults.^44^ Furthermore, the four countries focused on different settings, such as Spain on the educational setting, Portugal and the UK on the community setting and CZ on both settings.

The ability or skills of peer leaders can increase as the intervention progresses, as demonstrated in the present study. The best results in key competencies were found for the peer leaders from Spain and Portugal, where the selection of peer leaders was performed by people who were close to adolescents; conversely, in the other two countries, CZ and UK, the selection was made by pedagogical or communication experts who were not in contact with adolescents. Additionally, Spain and Portugal implemented similar interventions and leisure activities, whereas CZ was focused on personal experiences, and the UK was focused on the emotional aspect of lifestyles. Moreover, the number of hours of peer leader training differed among the countries. For example, Spain and CZ had scheduled training, whereas the UK and Portugal held training only when necessary.

Coach support is another important aspect of peer leader training. All of the countries claimed that the coaches were in constant contact with peer leaders, but the programmes in Spain and Portugal included personal contact weekly or biweekly throughout the intervention. This frequent contact might have resulted in promoting greater interest and confidence among the peer leaders and in enhancing the experience among the adolescent population. In contrast, frequent contact could have had negative effects on peer leader management because the peer leaders likely did not have sufficient freedom to develop some aspects on their own. However, other studies have supported the necessity of coach support during training for peer‐led adolescents and the implementation of projects.^45^, ^46^

In addition, the Spanish peer leaders designed part of the material used in the interventions, such as the infographic material, to support their activities, likely increasing their interest and motivation. Finally, the initial English level of the peer leaders from CZ and the UK was higher than that of the peer leaders from Spain or Portugal, which could be observed in the first exchange meeting, when the adolescent peer leaders from the EYTO project met in London. This fact could be a reason why the peer leaders from Spain and Portugal made additional efforts to use English throughout the project and had communication experts, thus improving communication tasks.

**Figure 2:**
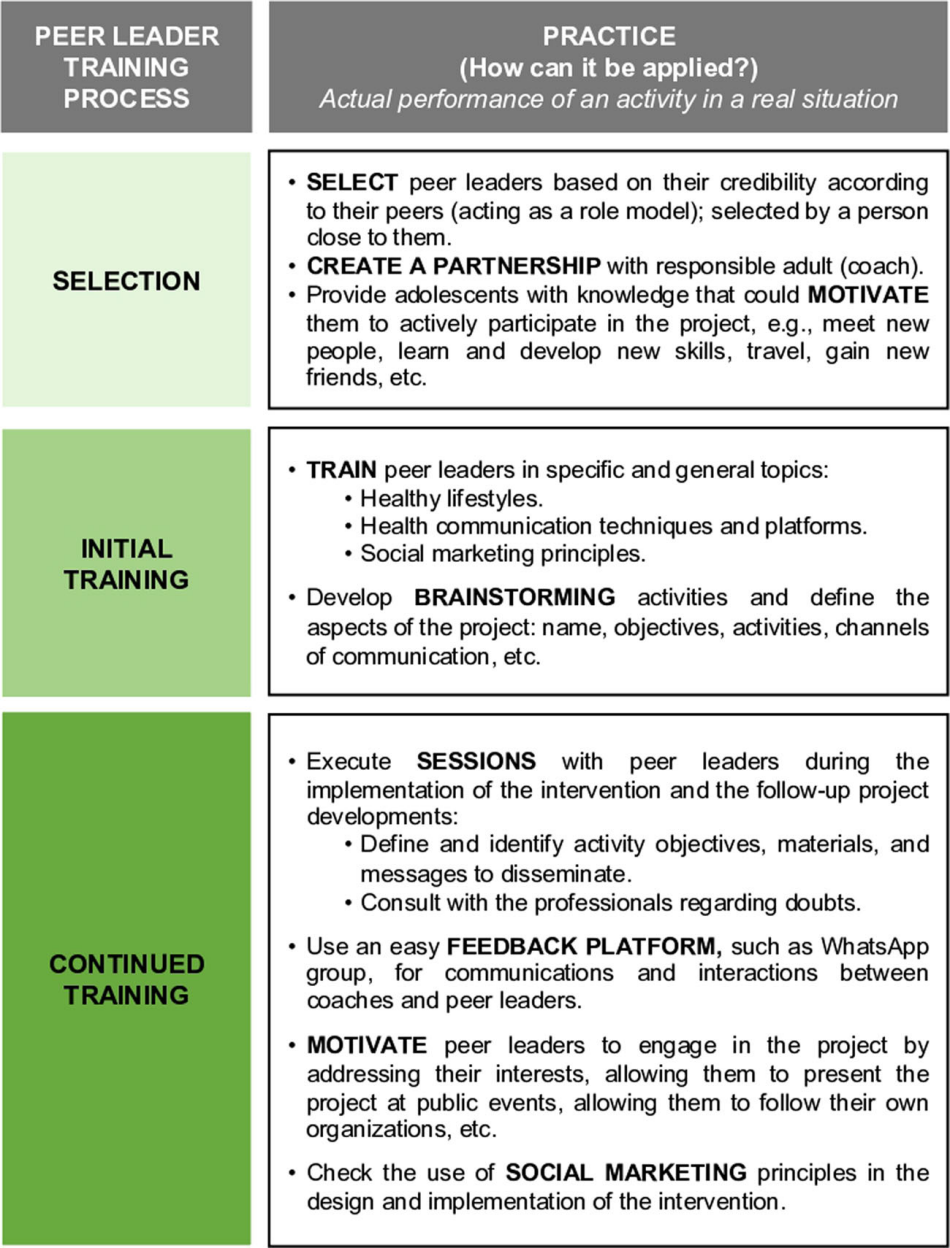
Recommendations for the peer leader training process to encourage healthy lifestyles among adolescents

**Figure 3:**
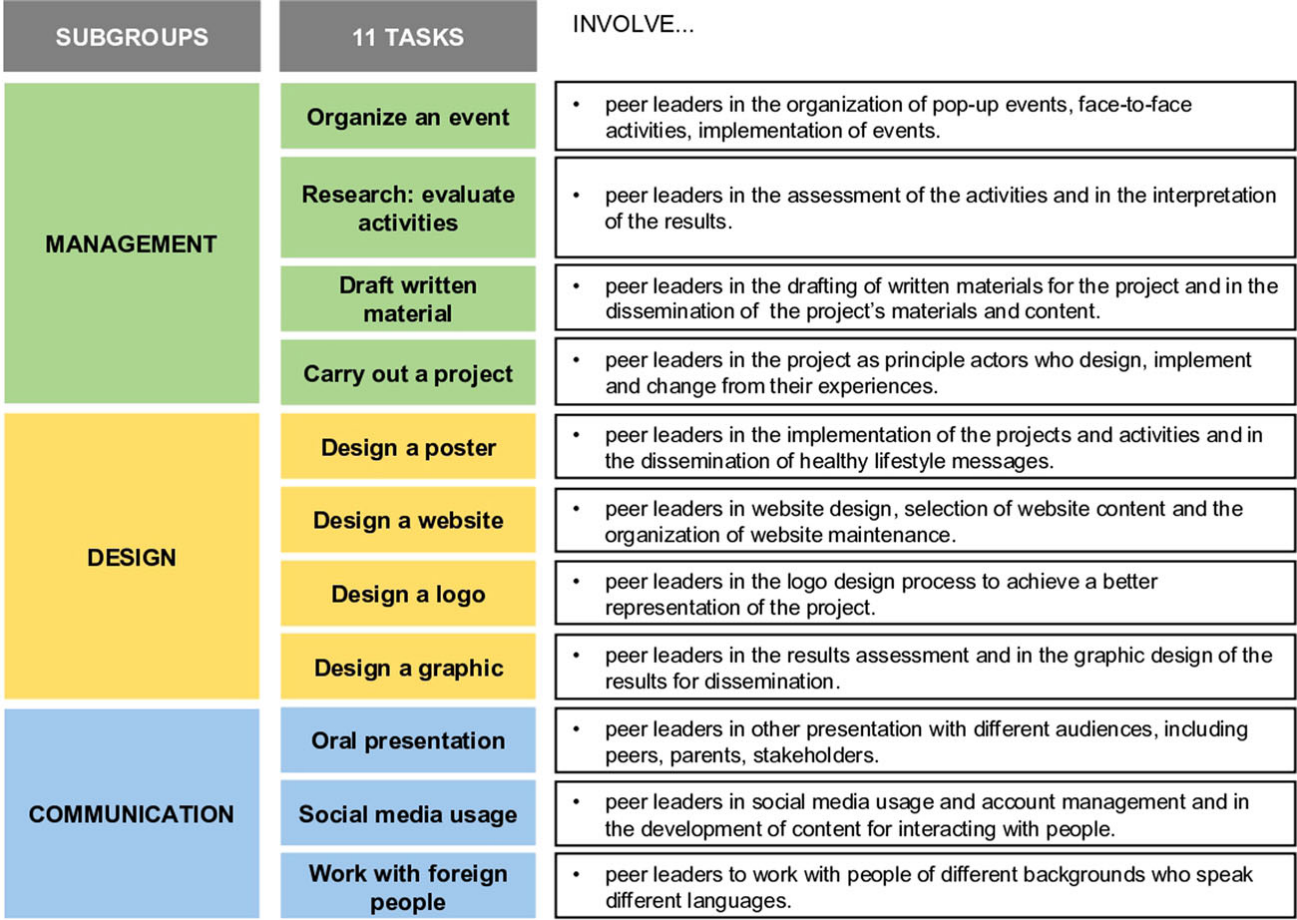
Specific suggestions for the peer leader training process to encourage the improvement of 11 tasks based on key competencies

Moreover, based on the EYTO peer leader training experience, we compiled recommendations for practices for stakeholders and researchers regarding the training of adolescents as peer leaders (Figure 2), as well as suggestions for improving the 11 tasks (Figure 3).

This study has certain limitations. First, since the number of peer leaders was small, it was difficult to obtain conclusive results. Thus, the findings on the confidence, experience and interest in developing and implementing these tasks among the three or five peer leaders per country should be verified through an intervention that includes a greater diversity of peer leaders from more diverse environments to evaluate the generalizability of the results. Moreover, the assessments of these tasks, as well as the methods of the assessments, should also be validated. Despite its limitations, the present study contributes to the research by presenting a novel methodology that has great potential in the field of health promotion. At the same time, the study serves as a guide to determine the aspects that should be strengthened in the future to identify the best methodology to obtain successful results.

In conclusion, the peer leaders improved their confidence in management tasks and their confidence and experience in communication tasks. Slight differences were detected in competencies improved by country, likely due to the dissimilarities among peer training applied. Recommendations for peer leader training were proposed, although these results should be verified with larger sample size.

## CONFLICT OF INTERESTS

Any of the authors declare conflicts of interest.

## ETHICS STATEMENT

The study participants provided informed consent (a form signed by the participants and by their parents/legal guardians).

## AUTHORS CONTRIBUTION

*The conception and design of the study, acquisition of data, analysis and interpretation of data*: Elisabet Llauradó, Magaly Aceves‐Martins, Jordi Prades‐Tena, Maria Besora‐Moreno, Ignasi Papell‐Garcia, Montse Giralt, Amy Davies, Lucia Tarro, Rosa Solà. *Drafting the article or revising it critically for important intellectual content*: Elisabet Llauradó, Magaly Aceves‐Martins, Jordi Prades‐Tena, Maria Besora‐Moreno, Ignasi Papell‐Garcia, Montse Giralt, Amy Davies, Lucia Tarro, Rosa Solà. *Final approval of the version to be submitted*: Elisabet Llauradó, Magaly Aceves‐Martins, Jordi Prades‐Tena, Maria Besora‐Moreno, Ignasi Papell‐Garcia, Montse Giralt, Amy Davies, Lucia Tarro, Rosa Solà. *Agreed to be accountable for all aspects of the work in ensuring that questions related to the accuracy or integrity of any part of the work are appropriately investigated and resolved*: Elisabet Llauradó, Magaly Aceves‐Martins, Jordi Prades‐Tena, Maria Besora‐Moreno, Ignasi Papell‐Garcia, Montse Giralt, Amy Davies, Lucia Tarro, Rosa Solà.

## Supporting information

Supporting information.

## Data Availability

The technical appendix and raw data set are available from the following authors upon request: elisabet.llaurado@urv.cat and lucia.tarro@urv.cat
