# Supplementary material for: Adolescents encouraging healthy lifestyles through a peer‐led social marketing intervention: Training and key competencies learned by peer leaders
Source: Health Expect. 2021 Dec 22;25(1):455–65. doi: 10.1111/hex.13406 (PMC8849247; doi:10.1111/hex.13406)
Supplement: Supplementary file 1 — Supporting information. [file HEX-25-455-s001.docx]

**Supplementary material**

Supplementary Table 1. Questionnaire

| **EUROPEAN YOUTH TACKLING OBESITY - CAMPAIGN CREATORS BASELINE DISCUSSION - STICKER TASK FINDINGS** | | | |
| --- | --- | --- | --- |
| **INSTRUCTION: Please indicated the total number in each cell** | | | |
| **1) EVER DONE THE ACTIVITY BEFORE: Coloured sticker activity (Please place the sticker in the box corresponding to the answer)** | **Green**  **(yes a lot)** | **Yellow**  **(yes slightly)** | **Red**  **(no)** |
| Organized or helped run an event |  |  |  |
| Carried out research |  |  |  |
| Designed a poster |  |  |  |
| Designed a website |  |  |  |
| Designed a logo |  |  |  |
| Done graphic design |  |  |  |
| Drafted written material to get across a key message to many people in a snappy or powerful way |  |  |  |
| Given a presentation to many people |  |  |  |
| Used social media to communicate a message to a wide range of people |  |  |  |
| Carried out a project with people you have never met before |  |  |  |
| Worked with people from other countries |  |  |  |
| **2) INTERESTED IN THE ACTIVITY: Coloured sticker activity (Please place the sticker in the box which corresponds to the answer)** | **Green**  **(yes a lot)** | **Yellow**  **(yes slightly)** | **Red**  **(no, not interested)** |
| Organized or helped run an event |  |  |  |
| Carried out research |  |  |  |
| Designed a poster |  |  |  |
| Designed a website |  |  |  |
| Designed a logo |  |  |  |
| Done graphic design |  |  |  |
| Drafted written material to get across a key message to many people in a snappy or powerful way |  |  |  |
| Given a presentation to many people |  |  |  |
| Used social media to communicate a message to a wide range of people |  |  |  |
| Carried out a project with people you have never met before |  |  |  |
| Worked with people from other countries |  |  |  |
| **2) WHETHER YOU HAVE THE CONFIDENCE AND SKILLS: Coloured sticker activity (Please place the sticker in the box which corresponds to the answer)** | **Green**  **(yes a lot)** | **Yellow**  **(yes slightly)** | **Red**  **(no)** |
| Organized or helped run an event |  |  |  |
| Carried out research |  |  |  |
| Designed a poster |  |  |  |
| Designed a website |  |  |  |
| Designed a logo |  |  |  |
| Done graphic design |  |  |  |
| Drafted written material to get across a key message to many people in a snappy or powerful way |  |  |  |
| Given a presentation to many people |  |  |  |
| Used social media to communicate a message to a wide range of people |  |  |  |
| Carried out a project with people you have never met before |  |  |  |
| Worked with people from other countries |  |  |  |

Supplementary Table 2. Peer leaders' experience, confidence, and interest in realizing specific tasks by country.

| **Tasks** | **Country** | **EXPERIENCE** | | | | | **CONFIDENCE** | | | | | **INTEREST** | | | | |
| --- | --- | --- | --- | --- | --- | --- | --- | --- | --- | --- | --- | --- | --- | --- | --- | --- |
|  |  | **Baseline** | | **End of study** | | ***p-value** | **Baseline** | | **End of study** | | ***p-value** | **Baseline** | | **End of study** | | ***p-value** |
|  |  | **n** | **%** | **n** | **%** |  | **n** | **%** | **n** | **%** |  | **n** | **%** | **n** | **%** |  |
| 1. Organize an event | Spain | 5 | 100 | 5 | 100 | ___ | 5 | 100 | 5 | 100 | ___ | 5 | 100 | 5 | 100 | ___ |
|  | UK | 1 | 33.3 | 2 | 66.7 | 1.00 | 3 | 100 | 3 | 100 | ___ | 3 | 100 | 3 | 100 | ___ |
|  | Portugal | 3 | 60 | 5 | 100 | 0.44 | 3 | 60 | 5 | 100 | 0.44 | 5 | 100 | 5 | 100 | ___ |
|  | CZ | 1 | 20 | 3 | 60 | 0.52 | 3 | 60 | 4 | 80 | 1.00 | 5 | 100 | 5 | 100 | ___ |
| 1. Research: evaluate activities | Spain | 2 | 40 | 5 | 100 | 0.17 | 5 | 100 | 5 | 100 | ___ | 5 | 100 | 5 | 100 | ___ |
|  | UK | 1 | 33.3 | 0 | 0 | 1.00 | 3 | 100 | 3 | 100 | ___ | 2 | 66.7 | 3 | 100 | 1.00 |
|  | Portugal | 3 | 60 | 5 | 100 | 0.44 | 3 | 60 | 5 | 100 | 0.44 | 2 | 40 | 1 | 20 | 1.00 |
|  | CZ | 0 | 0 | 0 | 0 | ___ | 1 | 20 | 5 | 100 | **0.048** | 3 | 60 | 4 | 80 | 1.00 |
| 1. Design a poster | Spain | 1 | 20 | 1 | 20 | 1.00 | 4 | 80 | 1 | 20 | 0.21 | 3 | 60 | 5 | 100 | 0.44 |
|  | UK | 1 | 33.3 | 2 | 66.7 | 1.00 | 3 | 100 | 3 | 100 | ___ | 1 | 33.3 | 3 | 100 | 0.40 |
|  | Portugal | 1 | 20 | 0 | 0 | 1.00 | 2 | 40 | 2 | 40 | 1.00 | 5 | 100 | 5 | 100 | ___ |
|  | CZ | 3 | 60 | 3 | 60 | 1.00 | 2 | 40 | 3 | 60 | 1.00 | 2 | 40 | 3 | 60 | 1.00 |
| 1. Design a website | Spain | 0 | 0 | 1 | 20 | 1.00 | 1 | 20 | 5 | 100 | **0.048** | 0 | 0 | 5 | 100 | **0.008** |
|  | UK | 0 | 0 | 0 | 0 | ____ | 0 | 0 | 2 | 66.7 | 0.40 | 2 | 66.7 | 3 | 100 | 1.00 |
|  | Portugal | 0 | 0 | 4 | 80 | **0.048** | 2 | 40 | 2 | 40 | 1.00 | 5 | 100 | 5 | 100 | ___ |
|  | CZ | 3 | 60 | 3 | 60 | 1.00 | 2 | 40 | 3 | 60 | 1.00 | 2 | 40 | 2 | 40 | 1.00 |
| 1. Design a logo | Spain | 0 | 0 | 1 | 20 | 1.00 | 1 | 20 | 1 | 20 | 1.00 | 5 | 100 | 5 | 100 | ___ |
|  | UK | 0 | 0 | 2 | 66.7 | 0.40 | 2 | 66.7 | 3 | 100 | 1.00 | 2 | 66.7 | 3 | 100 | 1.00 |
|  | Portugal | 1 | 20 | 5 | 100 | **0.048** | 2 | 40 | 5 | 100 | 0.17 | 5 | 100 | 5 | 100 | ___ |
|  | CZ | 2 | 40 | 4 | 80 | 0.52 | 1 | 20 | 3 | 60 | 0.52 | 5 | 100 | 2 | 40 | 0.17 |
| 1. Prepare a graphic | Spain | 3 | 60 | 1 | 20 | 0.52 | 0 | 0 | 1 | 20 | 1.00 | 0 | 0 | 5 | 100 | **0.008** |
|  | UK | 0 | 0 | 2 | 66.7 | 0.40 | 0 | 0 | 0 | 0 | ___ | 1 | 33.3 | 3 | 100 | 0.40 |
|  | Portugal | 0 | 0 | 1 | 20 | 1.00 | 0 | 0 | 2 | 40 | 0.44 | 5 | 100 | 1 | 20 | **0.048** |
|  | CZ | 2 | 40 | 4 | 80 | 0.52 | 1 | 20 | 2 | 40 | 1.00 | 1 | 20 | 3 | 60 | 0.52 |
| 1. Draft written material for dissemination to many people | Spain | 3 | 60 | 5 | 100 | 0.44 | 2 | 40 | 5 | 100 | 0.17 | 5 | 100 | 5 | 100 | ___ |
|  | UK | 2 | 66.7 | 3 | 100 | 1.00 | 3 | 100 | 3 | 100 | ___ | 2 | 66.7 | 3 | 100 | 1.00 |
|  | Portugal | 1 | 20 | 2 | 40 | 1.00 | 1 | 20 | 0 | 0 | 1.00 | 5 | 100 | 5 | 100 | ___ |
|  | CZ | 2 | 40 | 4 | 80 | 0.52 | 0 | 0 | 3 | 60 | 0.17 | 2 | 40 | 3 | 60 | 1.00 |
| 1. Give an oral presentation to a large audience | Spain | 5 | 100 | 5 | 100 | ___ | 5 | 100 | 5 | 100 | ___ | 5 | 100 | 5 | 100 | ___ |
|  | UK | 3 | 100 | 3 | 100 | ___ | 2 | 66.7 | 3 | 100 | 1.00 | 1 | 33.7 | 3 | 100 | 0.40 |
|  | Portugal | 0 | 0 | 5 | 100 | **0.008** | 1 | 20 | 4 | 80 | 0.21 | 1 | 20 | 5 | 100 | **0.048** |
|  | CZ | 5 | 100 | 5 | 100 | ___ | 2 | 40 | 3 | 60 | 1.00 | 2 | 40 | 4 | 80 | 0.52 |
| 1. Use social media to communicate a message | Spain | 0 | 0 | 5 | 100 | **0.008** | 1 | 20 | 5 | 100 | **0.048** | 5 | 100 | 5 | 100 | ___ |
|  | UK | 1 | 33.3 | 3 | 100 | 0.40 | 2 | 66.7 | 3 | 100 | 1.00 | 3 | 100 | 3 | 100 | ___ |
|  | Portugal | 0 | 0 | 1 | 20 | 1.00 | 2 | 40 | 1 | 20 | 1.00 | 5 | 100 | 2 | 40 | 0.83 |
|  | CZ | 2 | 40 | 4 | 80 | 0.52 | 1 | 20 | 4 | 80 | 0.21 | 2 | 40 | 4 | 80 | 0.26 |
| 1. Complete a project while working with strangers | Spain | 3 | 60 | 5 | 100 | 0.44 | 5 | 100 | 5 | 100 | ___ | 5 | 100 | 5 | 100 | ___ |
|  | UK | 3 | 100 | 2 | 66.7 | 1.00 | 2 | 66.7 | 3 | 100 | 1.00 | 2 | 66.7 | 3 | 100 | 1.00 |
|  | Portugal | 2 | 40 | 5 | 100 | 0.17 | 4 | 80 | 5 | 100 | 1.00 | 5 | 100 | 4 | 80 | 1.00 |
|  | CZ | 1 | 20 | 1 | 20 | 1.00 | 3 | 60 | 5 | 100 | 0.44 | 3 | 60 | 5 | 100 | 0.44 |
| 1. Collaborate with people from other countries | Spain | 2 | 60 | 5 | 100 | 0.44 | 5 | 100 | 5 | 100 | ___ | 5 | 100 | 5 | 100 | ___ |
|  | UK | 1 | 33.3 | 2 | 66.7 | 1.00 | 0 | 0 | 3 | 100 | 0.10 | 2 | 66.7 | 3 | 100 | 1.00 |
|  | Portugal | 0 | 0 | 0 | 0 | ___ | 2 | 40 | 5 | 100 | 0.17 | 5 | 100 | 5 | 100 | ___ |
|  | CZ | 5 | 100 | 5 | 100 | ___ | 5 | 100 | 5 | 100 | ___ | 5 | 100 | 5 | 100 | ___ |

UK: United Kingdom; CZ; Czech Republic

The results show only the percentage of adolescents who answered green to the question.

* Student Test —difference between baseline and end of study values.

The p-value of the significant results (p<0.05) is highlighted in bold.

Supplementary Table 3. Peer leaders' experience, confidence, and interest in management, design, and communication by country.

| **Subgroups** | **Country** | **EXPERIENCE** | | | | | **CONFIDENCE** | | | | | | **INTEREST** | | | | | |
| --- | --- | --- | --- | --- | --- | --- | --- | --- | --- | --- | --- | --- | --- | --- | --- | --- | --- | --- |
|  |  | **Baseline** | | **End of study** | | ***p-value** | **Baseline** | | **End of study** | | ***p-value** | | **Baseline** | | **End of study** | | ***p-value** | |
|  |  | **Mean** | **SD** | **Mean** | **SD** |  | **Mean** | **SD** | **Mean** | **SD** |  | **Mean** | | **SD** | **Mean** | **SD** |  |  |
| Management | Spain | 2.60 | 1.52 | 4.00 | 0 | 0.11 | 3.40 | 0.55 | 4.00 | 0 | 0.07 | 4.00 | | 0 | 4.00 | 0 | ___ |  |
|  | UK | 1.40 | 1.67 | 1.40 | 1.52 | 1.00 | 1.80 | 2.05 | 2.40 | 2.19 | 0.67 | 1.80 | | 2.05 | 2.40 | 2.19 | 0.67 |  |
|  | Portugal | 1.80 | 1.79 | 3.40 | 0.55 | 0.12 | 2.20 | 1.64 | 3.00 | 0 | 0.34 | 3.40 | | 0.55 | 3.00 | 0.71 | 0.35 |  |
|  | CZ | 0.80 | 1.30 | 1.60 | 1.14 | 0.33 | 1.40 | 1.34 | 3.40 | 0.89 | **0.02** | 2.60 | | 1.52 | 3.40 | 0.89 | 0.34 |  |
| Design | Spain | 0.80 | 0.84 | 0.80 | 1.79 | 1.00 | 1.20 | 1.10 | 1.60 | 1.34 | 0.62 | 1.60 | | 0.55 | 4.00 | 0 | **<0.001** |  |
|  | UK | 0.20 | 0.45 | 1.20 | 1.64 | 0.25 | 1.00 | 1.00 | 1.60 | 1.52 | 0.48 | 1.20 | | 1.79 | 2.40 | 2.19 | 0.37 |  |
|  | Portugal | 0.40 | 0.89 | 2.00 | 0.71 | **0.01** | 1.20 | 1.64 | 2.20 | 1.64 | 0.36 | 4.00 | | 0 | 3.20 | 0.45 | **0.02** |  |
|  | CZ | 2.00 | 2.00 | 2.80 | 1.79 | 0.52 | 1.20 | 1.79 | 2.20 | 2.05 | 0.44 | 2.00 | | 1.41 | 2.00 | 2.00 | 1.00 |  |
| Communication | Spain | 1.60 | 0.55 | 3.00 | 0 | **0.005** | 2.20 | 0.45 | 3.00 | 0 | **0.02** | 3.00 | | 0 | 3.00 | 0 | ___ |  |
|  | UK | 1.00 | 1.23 | 1.60 | 1.52 | 0.51 | 0.80 | 1.10 | 1.80 | 1.64 | 0.30 | 1.20 | | 1.30 | 1.80 | 1.64 | 0.54 |  |
|  | Portugal | 0 | 0 | 1.20 | 0.45 | **0.004** | 1.00 | 1.41 | 2.00 | 0.71 | 0.21 | 2.20 | | 0.45 | 2.40 | 0.55 | 0.55 |  |
|  | CZ | 2.40 | 0.55 | 2.80 | 0.45 | 0.24 | 1.60 | 0.89 | 2.40 | 0.89 | 0.20 | 1.80 | | 1.10 | 2.60 | 0.89 | 0.24 |  |

UK: United Kingdom; CZ; Czech Republic; SD: standard deviation

The results show only the percentage of adolescents who answered green to the question.

* Student Test —difference between baseline and end of study values.

The p-value of the significant results (p<0.05) is highlighted in bold.
